# Should All Translational Scientists Be Swifties?

**DOI:** 10.1016/j.jacbts.2024.02.004

**Published:** 2024-02-28

**Authors:** Douglas L. Mann

**Figure:**
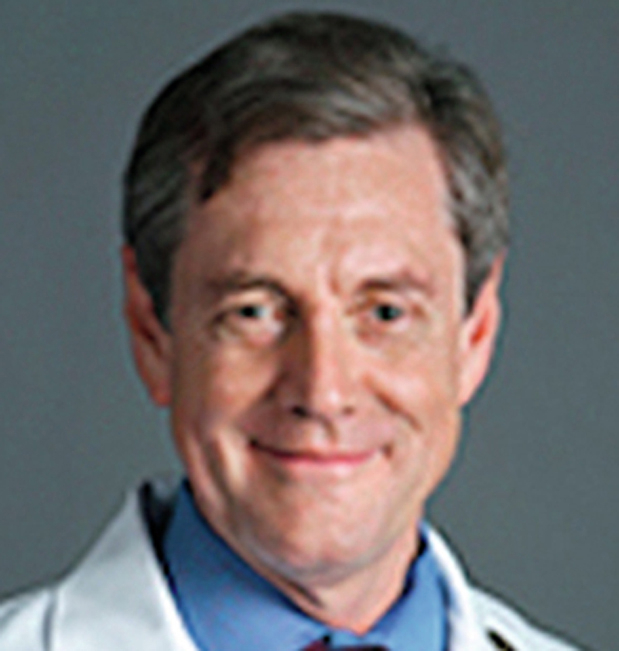

Over the past 9 years I have focused this Editor's Page on current issues related to cardiovascular translational science. Nonetheless, there are times when it is challenging to generate new ideas that will be of interest to the readership. Fortunately, I was able to find my inspiration for this editorial from the 2024 Grammys, where Taylor Swift received her unprecedented fourth Album of the Year award for *Midnights*. At age 34, Taylor Swift has received more Album of the Year awards than any other musical artist, including Frank Sinatra, Stevie Wonder, and Paul Simon, each of whom received 3 Grammy Album of the Year awards.

As someone who has tried to champion the field of translational science and the accomplishments of translational scientists, I thought about how I might be able tap into the excitement that Taylor Swift provides to her fans (aka Swifties) and bring that same level of energy to the readership of *JACC: Basic to Translational Science*.

Although I grew up with the music of Frank Sinatra in my house (my mom was a fan), and I greatly enjoyed the music of Stevie Wonder and Paul Simon, I knew I would need help with understanding how to capture the magic that Taylor Swift’s music brings to her audiences, insofar as I was completely unfamiliar with her musical canon. Therefore, I asked ChatGPT (v 4.0, OpenAI) to help me out with this Editor’s Page.

The rolling back-and-forth dialog that follows below represents a series of prompts from me (DLM) and replies from ChatGPT that were copied verbatim and have not been edited for grammar, style, or content.

**DLM:** Can you draft a 1,000-word essay on if Taylor Swift was a translational scientist, what would she sing about?

**ChatGPT:**

## Title: Harmonizing Science and Song: Taylor Swift as a Translational Scientist

In the realm of pop culture, Taylor Swift is renowned for her ability to capture the essence of human emotion through her lyrics. But what if this musical virtuoso ventured into the domain of translational science? What would she sing about? As we delve into this hypothetical scenario, we discover a harmonious fusion of scientific inquiry and lyrical expression, where Swift's melodies resonate with the complexities and wonders of scientific exploration.

## Verse 1: The Art of Discovery

In her songs, Taylor Swift often reflects on personal experiences and the journey of self-discovery. Similarly, as a translational scientist, she would likely draw inspiration from the process of scientific inquiry. Her lyrics might echo the thrill of uncovering new knowledge, the joy of experimentation, and the resilience required to overcome obstacles in the pursuit of truth.

In "The Art of Discovery," Swift celebrates the beauty of scientific exploration:

"In the laboratory of life, we paint with curiosity,

Mixing hues of hypothesis, with precision and clarity.

With each experiment, a canvas unfurls,

Revealing the mysteries of our world."

Through her music, Swift invites listeners to embark on a voyage of intellectual discovery, where the pursuit of knowledge becomes a poetic endeavor.

## Verse 2: Bridging Worlds

Translational science bridges the gap between research and practical application, seeking to translate scientific findings into tangible benefits for society. Taylor Swift, with her gift for storytelling, would undoubtedly explore the transformative power of scientific innovation in her songs.

In "Bridging Worlds," Swift sings of the profound impact of translational science:

"From bench to bedside, we forge a path,

Guided by compassion, we conquer wrath.

With every breakthrough, a bridge is built,

Connecting dreams to realities, woven without guilt."

Through her music, Swift illuminates the potential of science to bridge divides and improve lives, inspiring hope for a brighter future.

## Verse 3: The Rhythm of Collaboration

Collaboration lies at the heart of translational science, as researchers, clinicians, and patients come together to tackle complex challenges. Taylor Swift, known for her ability to collaborate with other artists, would likely explore the power of teamwork and collective effort in her songs.

In "The Rhythm of Collaboration," Swift celebrates the synergy of minds working in harmony:

"In the symphony of science, we find our tune,

A chorus of voices, rising 'neath the moon.

With each contribution, a melody is born,

Uniting hearts and minds, from dusk 'til morn."

Through her music, Swift champions the spirit of collaboration, reminding us that together, we can achieve great things.

## Verse 4: Embracing Uncertainty

Science is characterized by its embrace of uncertainty, as researchers grapple with unanswered questions and unexpected results. Taylor Swift, known for her introspective lyrics, would likely explore the themes of resilience and perseverance in the face of uncertainty.

In "Embracing Uncertainty," Swift reflects on the transformative power of adversity:

"In the labyrinth of life, we wander and we roam

Embracing uncertainty, we find our way home.

With every setback, a lesson learned,

Fueling our journey, 'til the tables turn."

Through her music, Swift inspires listeners to embrace life's uncertainties and emerge stronger on the other side.

## Conclusion: A Symphony of Science and Song

In the hypothetical world where Taylor Swift is a translational scientist, her music becomes a vehicle for exploring the wonders of scientific inquiry and the transformative power of innovation. Through her lyrics, she celebrates the joy of discovery, the impact of collaboration, and the resilience required to navigate the complexities of scientific exploration. In this harmonious fusion of science and song, Swift invites listeners to embark on a journey of intellectual discovery, where the pursuit of knowledge becomes a poetic endeavor. As we ponder this hypothetical scenario, we are reminded of the universal truths that bind humanity together—the quest for understanding, the power of collaboration, and the resilience of the human spirit.

**DLM:** When did you learn how to rhyme?

**ChatGPT:** As an AI language model developed by OpenAI, I don't "learn" in the same way humans do. My abilities, including rhyme generation, are a result of the vast amount of text data I've been trained on, which includes a wide range of poetry, lyrics, and other rhyming texts. Through this extensive training process, I've learned to recognize patterns in language and generate responses that often include rhymes when appropriate.

**DLM:** In January 2023 I wrote an editorial about ChatGPT 3.5 and translational medicine. Since that time I think you have grown smarter.

**ChatGPT**: Thank you for your kind words! It's certainly possible that advancements have been made since January 2023. The team at OpenAI is constantly working to improve and refine the capabilities of models like ChatGPT, aiming to make them more efficient and effective in understanding and generating human-like text. If you have any specific questions or topics you'd like to discuss regarding ChatGPT or translational medicine, feel free to let me know!

## An Editors’ Perspective on Generative AI

After the public release of ChatGPT 3.5, I wrote an article *for JACC: Basic to Translational Science*, where I prompted ChatGPT to discuss the pros and cons of AI in translational research.^1^ Although I was amazed by the blinding speed with which ChatGPT generated compelling arguments for and against the role of AI in translational research, I found that many of ChatGPT’s responses lacked “the type of nuanced insight that a person with in-depth knowledge of the field might provide. Indeed, the responses generated by the chatbot in response to my prompts were comparable to what an early career graduate student or first- or second-year medical student could generate after performing a 60- to 90-minute internet search using Google.” Recognizing that some experts estimate that AI systems capable of human-level thinking (referred to as AI singularity) would not be not be available for another 30-35 years,^2^ I noted in my article that that date for AI singularity was well beyond my tenure as Editor-in-Chief of *JACC: Basic to Translational Science*, and that I was “reasonably confident that I will be able to hold onto my editorial position and that an Editor-in-Chief chatbot will not replace me in the near term.”^1^ After reviewing ChatGPT’s hypothetical Taylor Swift song list especially “The Art of Discovery” and “Embracing Uncertainty,” I have undergone a damascene conversion with respect to my perspective on the current capabilities of generative AI. From my own personal experience as a translational investigator, ChatGPT’s hypothetical Taylor Swift lyrics (although a little hokey at times) did cover several recurring themes that translational investigators experience at some point in their careers. I also was feeling less confident about holding on to my job as the (human) Editor-in-Chief for *JACC: Basic to Translational Science*. So, I had to ask ChatGPT one last question.

**DLM:** Will generative AI replace journal editors?

**ChatGPT**: Generative AI has the potential to assist journal editors in various tasks such as content analysis, plagiarism detection, language polishing, and even potentially generating some types of content. However, the role of journal editors is multifaceted, involving not only quality control but also strategic decision-making, understanding the academic landscape, fostering scholarly discourse, and maintaining the integrity and reputation of the publication.

While generative AI can automate some aspects of the editorial process, it's unlikely to fully replace human editors in the foreseeable future. Human judgment, creativity, and understanding of context are essential in many editorial decisions, especially in fields where nuance, interpretation, and judgment calls are crucial.

**DLM:** Thank you. I needed that.
